# *In vivo* effect of fluoride combined with amoxicillin on enamel development in rats

**DOI:** 10.1590/1678-7757-2021-0171

**Published:** 2021-11-29

**Authors:** Juliana Feltrin-Souza, Silas Alves da Costa, Diego Girotto Bussaneli, Lourdes Santos-Pinto, Paulo Sérgio Cerri, Jaime Cury, Livia Tenuta, Rita de Cássia Loiola Cordeiro

**Affiliations:** 1 Universidade Federal do Paraná Departamento de Estomatologia Curitiba Paraná Brasil Universidade Federal do Paraná, Departamento de Estomatologia, Curitiba, Paraná, Brasil.; 2 Universidade Estadual Paulista Faculdade de Odontologia de Araraquara Departamento de Morfologia e Clínica Infantil Araraquara São Paulo Brasil Universidade Estadual Paulista, Faculdade de Odontologia de Araraquara, Departamento de Morfologia e Clínica Infantil, Araraquara, São Paulo, Brasil.; 3 Universidade de Campinas Faculdade de Odontologia de Piracicaba Departamento de Biociências São Paulo Brasil Universidade de Campinas, Faculdade de Odontologia de Piracicaba, Piracicaba, Departamento de Biociências, São Paulo, Brasil.; 4 University of Michigan School of Dentistry Restorative Sciences and Endodontics Department of Cariology Ann Arbor MI United States University of Michigan School of Dentistry, Restorative Sciences and Endodontics, Department of Cariology, Ann Arbor, MI, United States.

**Keywords:** Dental fluorosis, Rats, Amoxicillin, Enamel defect

## Abstract

Some evidence *in vitro* suggested that amoxicillin and fluoride could disturb the enamel mineralization. Objective: To assess the effect of amoxicillin and of the combination of amoxicillin and fluoride on enamel mineralization in rats. Methodology: In total, 40 rats were randomly assigned to four groups: control group (CG); amoxicillin group (AG - amoxicillin (500 mg/kg/day), fluoride group (FG - fluoridated water (100 ppm -221 mg F/L), and amoxicillin + fluoride group (AFG). After 60 days, the samples were collected from plasma and tibiae and analyzed for fluoride (F) concentration. The incisors were also collected to determine the severity of fluorosis using the Dental Fluorosis by Image Analysis (DFIA) software, concentration of F, measurements of enamel thickness, and hardness. The data were analyzed by ANOVA, Tukey’s *post-hoc* test, or Games-Howell post-hoc test (α=0.05). Results: Enamel thickness of the incisors did not differ statistically among the groups (p=0.228). Groups exposed to fluoride (AFG and FG) have higher F concentrations in plasma, bone and teeth than those not exposed to fluoride (CG and AG). The groups showed a similar behavior in the DFIA and hardness test, with the FG and AFG groups showing more severe fluorosis defects and significant lower hardness when compared with the AG and CG groups, with no difference from each other. Conclusion: The rats exposed to fluoride or fluoride + amoxicillin developed dental fluorosis, while exposure to amoxicillin alone did not lead to enamel defects.

## Introduction

The dental enamel development, known as amelogenesis, is a dynamic process resulting from the interplay between the epithelial and mesenchymal tissues.^1,2^ Even though genetic factors^3,4^ may play an important role in this process, extracellular disorders may cause changes in dental enamel.^5,6^ Developmental defects of enamel have been associated with recurrent health problems and medications in the first years of life, especially with conditions that cause high-grade fever.^6-8^ Amoxicillin is the main antibiotic used to treat bacterial infections in children. Amoxicillin has been previously demonstrated to affect the enamel formation in animals.^9-11^ Thus, whether the damage is caused by the disorder itself or by the medication use is still unclear, as both occur simultaneously and in close association.

The excessive chronic intake of fluoride may also affect amelogenesis, causing dental fluorosis with variable intensity.^12^ Besides the time of exposure to fluoride, its concentration, and bioavailability in blood plasma,^12,13^ the intensity of fluorosis has been associated in humans^14^ and rodents^15^ with genetic polymorphism as well as with hormonal,^16,17^ and environmental^18^ factors in humans. Animal studies suggest that enamel defects in rats exposed to a combination of fluorine and bisphenol-A were more severe than exposure to either agent alone.^19^ Exposure to lead also appears to exacerbate fluorotic lesions in rats.^20^ On the other hand, vitamin D and calcium associated with fluoride seem to protect against more severe forms of fluorosis in humans.^21^

Children chronically exposed to high dose of fluoride at the age between one and three years seem to be more prone to develop fluorosis.^5,12^ The mechanisms that clearly underlie this susceptibility remain unknown, but the increased risk of developing fluorosis was observed when fluoride intake was associated with treatments that used amoxicillin, especially in patients aged two years or less.^22,23^

Most experimental *in vivo* studies show dental effects in rodents exposed to amoxicillin.^24-27^ Some of them suggest amoxicillin can affect amelogenesis when administered in the prenatal period.^11,26,29^ An *in vitro* experiment showed that amoxicillin in combination with sodium fluoride can cause changes in amelogenesis.^28^ However, the effect of combining fluoride and amoxicillin on amelogenesis has not been assessed in controlled studies *in vivo*. Therefore, this *in vivo* study aimed to investigate the effect of amoxicillin and its combination with fluoride on amelogenesis in rats.

## Methodology

### Study groups and experimental design

The study was approved by the Animal Research Ethics Committee of Araraquara Dental School (CEUA- FOAr process no. 22/2012).

For estimation of the ideal sample size, a 95% confidence interval, 80% power, and previous parameters described in the literature were considered. The measures of central tendency and of dispersion used were the Dental Fluorosis by Image Analysis (DFIA)^30^ index and the hardness index.^31,32^ The ideal sample size ranged from four to 10 animals per group. In total, 40 male rats (*Rattus norvegicus, albinus, Holtzman*) aged 2-3 months and weighing around 200 g were selected. The rats were housed in polyethylene cages bedded on wood shavings, with at most two animals per cage, kept at the animal facility of Araraquara Dental School at controlled temperature (23±10ºC) and relative humidity (55±5%), artificial lighting with fluorescent lamp under a 12/12 h light/dark cycle, and fed Biotec pellets without fluoride (Biobase, Santa Catarina, Brazil) and water *ad libitum.*

After a 5-day adaptation period, the rats were randomly assigned to four groups of 10 rats each, with administration of the following substances every 24 h: CG (control group) – intragastric administration of deionized water in similar volumes to those of AG; AG - amoxicillin (Eurofarma Genéricos, São Paulo, Brazil), given intragastrically at the dose of 500 mg/kg of body weight, as oral suspension; FG – deionized water containing 100 ppm of fluoride *ad libitum, and* intragastric administration of deionized water in similar volumes to those of AG; AFG – intragastric administration of 500 mg/kg of amoxicillin per body weight, and daily administration of deionized water containing 100 ppm of fluoride *ad libitum*.

The substances were administered for 60 days, period during which incisors could fully develop again.^33^ Amoxicillin was prepared by diluting the content of the flask in deionized water at the concentration of 500 mg in 5 mL and that administration was done as described by Mihalaş, et al.^34^ (2016) and Souza, et al.^26^ (2016). Fluoride was prepared by diluting sodium fluoride in deionized water (221 mg NaF/L), obtaining 100 ppm of fluoride (100 mgF/L).^30,35^ The volume of water intake and the body mass of each animal were measured every 24 h.

After 60 days, the rats were euthanized with an overdose of 10% ketamine hydrochloride (Cetamin®- Syntec do Brasil Ltda, São Paulo, Brazil) in combination with 2% xylazine hydrochloride (Xilazin®- Syntec do Brasil Ltda, São Paulo, Brazil). Blood was collected by cardiac puncture using vacutainer blood collection tubes containing heparin as anticoagulant (BD Vacutainer Blood Collection Tubes, SST II Plus; BD Biosciences, New Jersey, USA). Upper and lower incisors were extracted and one tibia was removed from each animal, cleaned, coded for blind analysis, and stored at 60 °C for 24 h.

### Photographic analysis

In total, 10 lower incisors from each group were photographed and analyzed by the quantitative method known as Dental Fluorosis by Image Analysis (DFIA).^30^ Before being photographed, the incisors were fixed on paraffin-embedded slides, positioning the buccal surface at the incisal edge of the crown parallel to the macro lens (Medical 100 Macro, Canon, New York, USA), which was positioned and vertically attached 7 cm above the tooth. The standardized images were made with a digital camera (EOS Rebel XTI, Canon, New York, USA). An area of 690 × 170 pixels was selected from the image, converted to a grayscale, with dark and light areas, and analyzed by a software program specifically developed for that. The DFIA index was estimated by the difference between I_H_ (mean of pixels with the highest intensity, corresponding to the dark areas in the original image) and I_L_ (mean of pixels with the lowest intensity, corresponding to light-colored areas in the original image). The DFIA index was proposed by Catani, et al.^30^ (2010). The higher the index, the more severe the fluorosis.

### Analysis of enamel thickness

In total, 10 upper incisors from each group were transversely sectioned in three regions (incisal, medial, and apical) using a diamond bur at high speed. Each region was fixed on an acrylic resin block, with the cross-sectional tooth surface parallel to the horizontal plane. Three enamel thickness measurements were made in each region using 10× magnification on a microhardness tester (Future Tech, FM-7 model, Kawasaki, Japan) coupled to FM-ARS software.

### Hardness analysis

In total, ten upper incisors from each group were embedded on acrylic resin discs with their long axes parallel to the disc surface (Arotec EMB-30, Arotec Ind. Com. LTDA, São Paulo, Brazil). Subsequently, they were abraded up to the center of the tooth using 400-grit wet-dry sandpapers (Arotec APL-4, Arotec Ind. Com. LTDA, São Paulo, Brazil) and polished with higher-number sandpaper grits (600 to 1200) and then with a felt polishing wheel soaked in a 1-μm diamond suspension. Enamel hardness was measured by a microhardness tester (Future Tech, FM-7 model, Kawasaki, Japan) and Knoop indenter, coupled to FM-ARS software, in three tooth regions (incisal, medial, and apical). Each region was assessed at five depths from the outer surface of the enamel (10 μm, 20 μm, 30 μm, 50 μm, and 70 μm). The Knoop indenter was applied using the axial axis of the diamond parallel to the outer surface of the enamel with a load of 25 g for 5 s. Three indentations were performed at each depth and the mean of each depth was estimated. The data were expressed in Knoop hardness number (KHN), that were result from measurements performed by the microhardness tester using the length of the longest diagonal of the diamond (d), the constant related to the frame formed by the base of the indenter (C) and the load applied to the indentation (c), using the formula: $\mathrm{KHN}=C.c/d^{2}$.

### Fluoride concentration in blood plasma

The plasma samples were obtained after centrifugation at 2,000 g for 10 min. One milliliter of each sample was buffered with TISAB III (Orion Research, Massachusetts, USA) and subjected to analysis of fluoride concentration using an ion-selective electrode (Orion Research 96-09; Orion Research Incorporated, Massachusetts, USA) coupled to a potentiometer (Orion Research EA 940; Orion Research Incorporated, Massachusetts, USA). Prior to the analyses, the electrode was calibrated by a standard curve for fluoride concentration. The results were expressed as μg F/mL of plasma.

### Fluoride concentration in bone specimens

Ten tibiae from each group were dried at 60 °C for 24 h, transversely sectioned into 5-mm slices in the diaphysis area. A section of each specimen was powdered, sifted to obtain particles between 1 mm and 140 μm, and placed in an oven for another 24 h. Ten milligrams (±0.01 mg) of powder was transferred to test tubes containing 0.5 M HCl and shaken for 1 h. At the end of this period, the tubes were centrifuged, the supernatant was collected, with addition of 1.0 mL of TISAB II (1.0 M acetate buffer, pH 5.0 with 1.0 M NaCl and 0.4% CDTA), containing 0.5 M NaOH, followed by agitation. Fluoride concentration was determined using an ion-selective electrode (Orion Research 96-09; Orion Research Incorporated, Massachusetts, USA) coupled to a potentiometer (Orion Research EA 940; Orion Research Incorporated, Massachusetts, USA). The results were expressed as μg of F/g of bone.

### Fluoride concentration in teeth

Ten lower incisors from each group were transversely sectioned into three segments (apical, medial, and incisal), placed in an oven at 60 ºC and weighed separately thereafter, powdered, and sifted to obtain particles between 140 μm and 1,000 μm, and placed in an oven for another 24 h. Subsequently, 5 mg (±0.01 mg) of powder was transferred to test tubes containing 0.5 M HCl and shaken for 1 h. At the end of this period, the tubes were centrifuged and the supernatant was collected for quantification of fluoride, as described for the concentration of fluoride in rat tibiae.

### Statistical methods

Analyses of variance (ANOVA) were performed using Statistica version 8.0 (StatSoft Inc, Oklahoma, USA). When differences were observed between the groups, multiple comparisons of the means were made by Tukey’s test or by the Games-Howell test, deemed appropriate in the presence of heterogeneity of variance. The significance level was set as α=0.05. The relationship between surface depth and hardness was determined by linear regression procedures, computing the angular coefficient, which represents the variation in hardness units (KHN) by unit of depth, and by the linear regression coefficient (r).

## Results

A total of 40 rats (10 per group) were assessed. No statistical difference (ANOVA: p>0.05) was observed between the group means for water intake (42.1±5.6 mL) and for body mass (309.8±64.8 mg) among the rats included in the study.

**Figure 1:**
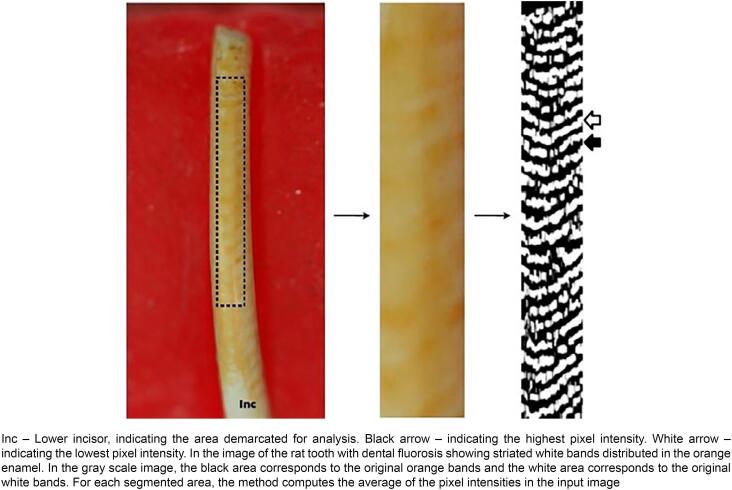
Schematic diagram of DFIA index for quantification of fluorosis

Table 1 shows the means and standard deviations of enamel thickness (in μm) and of the DFIA index, which determine the severity of fluorosis, according to the experimental groups assessed. No significant differences were found between the means obtained for enamel thickness between the groups. On the other hand, the means of DFIA for the groups exposed to fluoride (FG and AFG) presented significantly higher values than control (CG) or amoxicillin (AG) groups. Exposure to amoxicillin (AG) alone or with fluoride (AFG) did not influence the DFIA values. Figure 1 shows the diagram of DFIA index for quantification of fluorosis through the differences between the dark and light areas.

**Table 1:** Mean (SD) of enamel thickness (in μm) and of the DFIA index

Group	Enamel thickness*	DFIA**
CG	117.8 (9.5)	1.3 (0.9)^a^
AG	123.1 (10.0)	1.8 (1.0)^ab^
FG	122.2 (9.7)	4.5 (1.4)^c^
AFG	119.4 (9.0)	3.2 (1.9)^bc^

* No significant difference between the means of enamel thickness (ANOVA: p>0.05).

** Means of DFIA followed by identical letters are not significantly different according to Tukey’s test (p>0.05).

Table 2 shows the means and standard deviations of Knoop hardness, angular coefficients, and correlation coefficients of two linear regressions for depths of 10 to 30 μm. The groups exposed to fluoride (AFG and FG) had significantly lower mean hardness than that of non-exposed groups (AG and CG) at all depths. We can observe that the fluorosis was more severe on the outer enamel surface (10 μm), since the results are the same and greater for depths from 20 to 70 μm. Regarding exposure to amoxicillin, the enamel hardness values from rats only exposed to amoxicillin (AG) did not differ from the control group (CG), and are similar to the groups exposed to fluoride and amoxicillin (AFG) and only fluoride (FG).

**Table 2:** Mean (SD) of Knoop hardness (KHN). Estimates of angular coefficient (AC) and of standard error (SE): AC(SE) and correlation coefficient (r)

Group	Depth (μm)*					AC (SE)**	r***
	10	20	30	50	70		
CG	148.1 (47.6)^c^	233.6 (43.2)^b^	306.3 (46.8)^b^	354.3 (29.6)^b^	352.2 (31.3)^b^	7.9 (1.0)	0.83
AG	130.5 (35.0)^bc^	202.5 (31.2)^b^	271.8 (29.1)^b^	336.5 (22.0)^b^	336.6 (31.9)^b^	7.1 (0.7)	0.89
FG	79.5 (18.4)^a^	114.0 (23.9)^a^	158.7 (54.3)^a^	191.4 (46.0)^a^	179.1 (47.0)^a^	4.0 (0.8)	0.69
AFG	102.5 (22.5)^ab^	145.6 (27.9)^a^	211.3 (54.8)^a^	226.0 (43.4)^a^	212.3 (44.7)^a^	5.4 (0.9)	0.78

* Group means in the columns followed by identical letters are not significantly different according to Tukey’s test (p>0.05). The result is the same for the depths of 20 to 70 mm.

** Linear regression at 10 to 30 μm.

*** Linear correlation coefficient (r: p<0.05).

**Figure 2:**
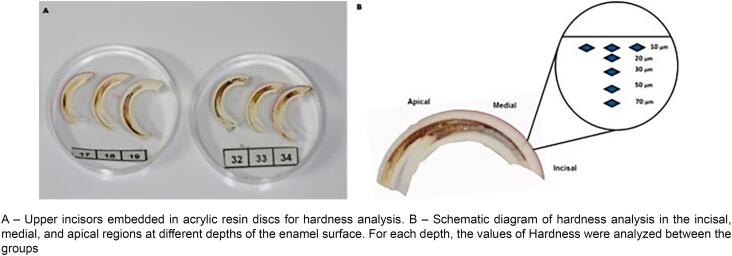
Rat teeth organized for hardness analysis

The regressions show significant linear increase in hardness at approximately 10 μm to 30 μm in groups not exposed to fluoride (CG and AG), with an increase by 7.9 and 7.1 KHN (Knoop hardness number) per μm, compared to an increase by 4.0 and 5.4 KHN per μm in the FG and AFG, respectively. Figure 2 shows the area of the three indentations that were performed, namely: incisal, medial, and apical regions.

Table 3 shows the analysis of variance, indicating significant difference between the mean fluoride concentrations in plasma, bone, and teeth. Groups exposed to fluoride (AFG and FG) have higher concentrations than those not exposed to fluoride (CG and FG) with no effect of amoxicillin on F concentrations.

**Table 3:** Mean (SD) concentration of F, expressed as μg/g of bone or tooth and as μg/mL of plasma

Group*	Blood plasma	Bone	Tooth
CG	0.02 (0.01)^a^	97.3 (20,5)^a^	35.6 (10.0)^a^
AG	0.05 (0.04)^a^	81.6 (27,7)^a^	45.6 (20.0)^a^
FG	0.38 (0.15)^b^	2250.3 (270,7)^b^	1907.0 (732.8)^b^
AFG	0.25 (0.13)^b^	2243.4 (470,3)^b^	2560.2 (894.7)^b^

* Group means in the column followed by identical letters did not show any differences in the Games-Howell test (p>0.05).

## Discussion

This *in vivo* study showed the effect of chronic exposure to fluoride and to amoxicillin on dental enamel. To the best of our knowledge, this is the first animal study that compared the effect of fluoride alone and of fluoride + amoxicillin on amelogenesis, considering previous hypotheses described in the literature.^23,28^ The methods presented herein allowed detecting dental fluorosis caused by chronic exposure to excessive fluoride. The methods applied in this study by DFIA index and hardness presented compatible results according to the experimental groups, validating the study model to the fluoride exposure.

The DFIA method was validated to detect the increase in the severity of fluorosis, determining the difference between light-colored and orange striated patterns observed in the exposure to fluoride.^30^ Our findings showed that high levels of fluoride caused dental fluorosis in rats, as observed by Catani, et al.^30^ (2010). Although the literature showed that amoxicillin can exert some influence on fluorosis,^24,28^ this study showed no differences between the control group and the group exposed to amoxicillin, despite the high doses of amoxicillin. One possible explanation could be that the rat strain used in this study is not sensitive to 500 mg amoxicillin/kg.

The rats exposed to fluoride presented severe fluorosis in the image analysis (DFIA index) and in surface hardness test, as expected, since the model for the induction of dental fluorosis in rodents used in this study is already known.^35^ The severity of fluorosis can be quantified and correlated with the level of injury caused by fluoride based on its plasma concentration.^36^ In this study, the analysis of fluoride on plasma and bone was performed to demonstrate the experiment’s validity. Bone tissue presents a highly dynamic behavior, with a constant rate of remodeling, and, therefore, fluoride exposure can affect the bone composition, its three-dimensional structure, and bone cells.^39^ Thus, the concentration of fluoride in bone tissue was higher than in the enamel. This can be explained by the higher bone metabolism of growing animals, since a higher proportion of fluoride is retained in calcified tissues in young people due to their high rates of remodeling bone.^40^ The effect of amoxicillin on F concentrations regardless of the tissue considered was not found.

Hardness values obtained for those groups treated with fluoride, either with or without association with amoxicillin (FG and AFG), showed severe fluorosis are related to high fluoride concentrations in the plasma, bone, and teeth, corroborating the findings of other authors.^30,36^ Moreover, after fluoride is absorbed, it remains in the bone tissue for a while and, consequently, fluoride is released into the bloodstream and locally to the tissues contiguous with the enamel in the mineralization stage, possibly increasing the severity of fluorosis.^12^

Considering the hardness results, our findings show a significant linear increase in hardness at the depths of 10 to 30 μm in all groups, suggesting that exposure to fluoride caused changes in the enamel, which were more prominent at the first 10 μm of the enamel surface. These findings corroborate those observed *in vivo* by Shinoda^31^ (1975), who described a reduction in enamel surface hardness at 10 μm caused by exposure to fluoride, demonstrating that this could be related to changes in the size, shape, and quality of enamel rods.

Amoxicillin is an antibiotic widely used in the treatment of bacteremia, and its most frequent side effects are related to dysregulation of the gastrointestinal tract. Studies in humans also point to the possible interference of amoxicillin on tooth enamel. Laisi, et al.^24^ (2009) reported that 16.3% of preschool children with molar incisor hypomineralization (MIH) used antibiotics during the first year of life, particularly amoxicillin. This finding was corroborated by Wuollet, et al.^44^ (2016), who found that children exposed to amoxicillin in the first 3 years had 2.58 times the risk of developing MIH. However, these studies are retrospective and influenced by confounders between the exposure and the outcome.

The 500 mg/kg daily dose of amoxicillin ministered in this study was estimated by simulating the exposure of infants/children to amoxicillin in their first years of life, according to the American Academy of Pediatrics.^37^ The total daily dose was ministered once a day, conveniently, since the effects on tooth development seem similar when the total daily dose was divided into 3 times a day^10^ or in a single dose.^38^

The amoxicillin did not influence on the enamel hardness and thickness in this study. No significant difference was observed between the groups exposed to fluoride + amoxicillin (AFG) and the fluoride group (FG) on the DFIA and hardness, as well as no difference between the AG and CG groups. Therefore, we suggest that exposure to amoxicillin did not exhibited interaction on the effect of fluoride on *in vivo* dental enamel. These data are not in accordance with other previous published data.

In animals’ study, Gottberg, et al.^11^ (2014) observed that the side effect caused by amoxicillin on dental enamel was dose-dependent in rodents. Souza, et al.^26^ (2016) have evidenced in a histological analysis that, during the secretory stage of the enamel matrix (seven days), exposure to amoxicillin reduced enamel thickness, but in the subsequent stage, at the end of secretion and at the outset of mineralization (12 days), this reduction in thickness was not observed between the exposed and non-exposed groups, thus suggesting a one-off effect of amoxicillin or a possible change in the secretion rate of the enamel matrix, which was later restored. Our results do not show statistically significant effects of amoxicillin on dental fluorosis in Holtzman rats, in accordance with de Souza, et al.^25^ (2020) who used the same rat strain and observed none effect of amoxicillin on the enamel mineralization.

Regarding enamel thickness, no difference among the groups was identified. The exposure to fluoride leads to changes in enamel mineralization, causing enamel fluorosis without morphological changes in the secretory stage, which is in line with the assumptions about the pathogenesis of dental fluorosis.^30,41,42^ However, *in vitro*^28^ and *in vivo*^24,26^ studies have shown that exposure to amoxicillin, to fluoride, or to both can affect enamel thickness during the initial secretory stage of enamel matrix formation. These controversial results should be analyzed in future studies that should also analyze the bioavailability of amoxicillin in the organism of the animal and the possible intestinal environment of absorption of the drug, as previously suggested by Chesa-Jiménez, et al.^43^ (1994).

Previous studies also demonstrate that some endogenous factors^15^ and exogenous molecules^21^ may modulate fluoride effects on dental enamel underlying the necessity for further studies with dose-response effects on various strains with careful considerations to external conditions. In rodents, the association between fluoride and bisphenol-A seems to increase severity of fluorosis in rats.^19^

## Conclusion

We conclude that chronic exposure to fluoride caused significant changes in dental enamel, compatible with the plasma concentrations of fluoride and with the reduction of enamel hardness, especially at the first 10 μm. Amoxicillin associated to fluoride did not influence the severity of fluorosis. Moreover, the exposure to amoxicillin alone has not affected amelogenesis in our rat model.

## References

[1] Gibson CW. The amelogenin proteins and enamel development in humans and mice. J Oral Biosci. 2011;53(3):248-56. doi: 10.2330/joralbiosci.53.248 10.2330/joralbiosci.53.248 PMC3247901 22215951

[2] Lu Y, Papagerakis P, Yamakoshi Y, Hu JC, Bartlett JD, Simmer JP. Functions of KLK4 and MMP-20 in dental enamel formation. Biol Chem. 2008;389(6):695-700. doi: 10.1515/BC.2008.080 10.1515/BC.2008.080 PMC2688471 18627287

[3] Alaluusua S. Aetiology of molar-incisor hypomineralisation: a systematic review. Eur Arch Paediatr Dent. 2010;11(2):53-8. doi: 10.1007/BF03262713 10.1007/BF03262713 20403298

[4] Hu JC, Chun YH, Al Hazzazzi T, Simmer JP. Enamel formation and amelogenesis imperfecta. Cells Tissues Organs. 2007;186(1):78-85. doi: 10.1159/000102683 10.1159/000102683 17627121

[5] Evans RW, Stamm JW. An epidemiologic estimate of the critical period during which human maxillary central incisors are most susceptible to fluorosis. J Public Health Dent. 1991;51(4):251-9. doi: 10.1111/j.1752-7325.1991.tb02223.x 10.1111/j.1752-7325.1991.tb02223.x 1941778

[6] Beentjes VE, Weerheijm KL, Groen HJ. Factors involved in the aetiology of molar-incisor hypomineralisation (MIH). Eur J Paediatr Dent. 2002;3(1):9-13. 12871011

[7] Arrow P. Risk factors in the occurrence of enamel defects of the first permanent molars among schoolchildren in Western Australia. Community Dent Oral Epidemiol. 2009;37(5):405-15. doi: 10.1111/j.1600-0528.2009.00480.x 10.1111/j.1600-0528.2009.00480.x 19694775

[8] Crombie F, Manton D, Kilpatrick N. Aetiology of molar-incisor hypomineralization: a critical review. Int J Paediatr Dent. 2009;19(2):73–83. doi: 10.1111/j.1365-263X.2008.00966.x 10.1111/j.1365-263X.2008.00966.x 19250392

[9] Ciarrocchi I, Masci C, Spadaro A, Caramia G, Monaco A. Dental enamel, fluorosis and amoxicillin. Pediatr Med Chir. 2012;34(3):148-54. doi: 10.4081/pmc.2012.84 10.4081/pmc.2012.84 22966729

[10] Kameli S, Moradi-Kor N, Tafaroji R, Ghorbani R, Farzadmnesh H, Sameni H. Effects of amoxicillin on the structure and mineralization of dental enamel and dentin in wistar rats. Front Dent. 2019;16(2):130-5. doi: 10.18502/fid.v16i2.1364 10.18502/fid.v16i2.1364 PMC6874840 31777854

[11] Gottberg B, Berne J, Quinonez B, Solorzano E. Prenatal effects by exposing to amoxicillin on dental enamel in wistar rats. Med Oral Patol Oral Cir Bucal. 2014;19(1):e38-43. doi: 10.4317/medoral.18807 10.4317/medoral.18807 PMC3909430 24121904

[12] Aoba T, Fejerskov O. Dental fluorosis: chemistry and biology. Crit Rev Oral Biol Med. 2002;13(2):155-70. doi: 10.1177/154411130201300206 10.1177/154411130201300206 12097358

[13] Patil MM, Lakhkar BB, Patil SS. Curse of fluorosis. Indian J Pediatr. 2018;85(5):375-83. doi: 10.1007/s12098-017-2574-z 10.1007/s12098-017-2574-z 29297143

[14] Duran-Merino D, Molina-Frechero N, Sánchez-Pérez L, Nevárez-Rascón M, González-González R, Tremillo-Maldonado O, et al. ENAM gene variation in students exposed to different fluoride concentrations. Int J Environ Res Public Health. 2020;17(6):1832. doi: 10.3390/ijerph17061832 10.3390/ijerph17061832 PMC7143052 32178265

[15] Charone S, Küchler EC, Leite AL, Fernandes MS, Pelá VT, Martini T, et al. Analysis of polymorphisms in genes differentially expressed in the enamel of mice with different genetic susceptibilities to dental fluorosis. Caries Res. 2019;53(2):228-33. doi: 10.1159/000491554 10.1159/000491554 30149392

[16] Dalledone M, Cunha AS, Ramazzotto LA, Pecharki GD, Nelson-Filho P, Scariot R, et al. Estrogen receptor gene is associated with dental fluorosis in Brazilian children. Clin Oral Investig. 2019;23(9):3565-70. doi: 10.1007/s00784-018-2778-2 10.1007/s00784-018-2778-2 30539292

[17] Priyanka D, Vandana K. Oestrogen receptor Rsa I gene polymorphism in osteoporosis periodontitis patients with or without dental fluorosis. Indian J Med Res. 2019;149(3):364-8. doi: 10.4103/ijmr.IJMR_1821_16 10.4103/ijmr.IJMR_1821_16 PMC6607811 31249201

[18] Akuno MH, Nocella G, Milia EP, Gutierrez L. Factors influencing the relationship between fluoride in drinking water and dental fluorosis: a ten-year systematic review and meta-analysis. J Water Health. 2019;17(6):845-62. doi: 10.2166/wh.2019.300 10.2166/wh.2019.300 31850893

[19] Jedeon K, Houari S, Loiodice S, Thuy TT, Le Normand M, Berdal A, et al. Chronic exposure to bisphenol A exacerbates dental fluorosis in growing rats. J Bone Miner Res. 2016;31(11):1955-66. doi: 10.1002/jbmr.2879 10.1002/jbmr.2879 27257137

[20] Leite GA, Sawan RM, Teófilo JM, Porto IM, Sousa FB, Gerlach RF. Exposure to lead exacerbates dental fluorosis. Arch Oral Biol. 2011;56(7):695-702. doi: 10.1016/j.archoralbio.2010.12.011 10.1016/j.archoralbio.2010.12.011 21269604

[21] Patel PP, Patel PA, Zulf MM, Yagnik B, Kajale N, Mandlik R, et al. Association of dental and skeletal fluorosis with calcium intake and serum vitamin D concentration in adolescents from a region endemic for fluorosis. Indian J Endocrinol Metab. 2017;21(1):190-5. doi: 10.4103/2230-8210.196013 10.4103/2230-8210.196013 PMC5240064 28217521

[22] Hong L, Levy SM, Warren JJ, Dawson D V, Bergus GR, Wefel JS. Association of amoxicillin use during early childhood with developmental tooth enamel defects. Arch Pediatr Adolesc Med. 2005;159(10):943-8. doi: 10.1001/archpedi.159.10.943 10.1001/archpedi.159.10.943 16203939

[23] Hong L, Levy SM, Warren JJ, Broffitt B. Amoxicillin use during early childhood and fluorosis of later developing tooth zones. J Public Health Dent. 2011;71(3):229-35. PMC4556648 21972463

[24] Laisi S, Ess A, Sahlberg C, Arvio P, Lukinmaa P-L, Alaluusua S. Amoxicillin may cause molar incisor hypomineralization. J Dent Res. 2009;88(2):132-6. doi: 10.1177/0022034508328334 10.1177/0022034508328334 19278983

[25] Feltrin-Souza J, Jeremias F, Alaluusua S, Sahlberg C, Santos-Pinto L, Jernvall J, et al. The effect of amoxicillin on dental enamel development *in vivo*. Braz Oral Res. 2020;34:e116. doi: 10.1590/1807-3107bor-2020.vol34.0116 10.1590/1807-3107bor-2020.vol34.0116 32901731

[26] Souza JF, Gramasco M, Jeremias F, Santos-Pinto L, Giovanini AF, Cerri PS, et al. Amoxicillin diminishes the thickness of the enamel matrix that is deposited during the secretory stage in rats. Int J Paediatr Dent. 2016;26(3):199-210. doi: 10.1111/ipd.12184 10.1111/ipd.12184 26148818

[27] Gao J, Li X, Gao L, Chen H, Baras BH, Liu X, et al. Effects of applying amoxicillin in juvenile mice on enamel mineralization and the expression of kallikrein-related peptidase 4 and tight junction proteins in ameloblasts. Int J Mol Med. 2020;46(1):179-90. doi: 10.3892/ijmm.2020.4598 10.3892/ijmm.2020.4598 PMC7255463 32626909

[28] Sahlberg C, Pavlic A, Ess A, Lukinmaa P-L, Salmela E, Alaluusua S. Combined effect of amoxicillin and sodium fluoride on the structure of developing mouse enamel *in vitro*. Arch Oral Biol. 2013;58(9):1155-64. doi: 10.1016/j.archoralbio.2013.03.007 10.1016/j.archoralbio.2013.03.007 23601745

[29] Mostafa H, Shehata F, Omar S, Kawana K. The effect of amoxicillin on the secretory stage of amelogenesis in rats. Alexandria Dent J. 2020;45(1):34-8. doi: 10.21608/adjalexu.2020.79929

[30] Catani DB, Tenuta LM, Andaló FA, Cury JA. Fluorosis in rats exposed to oscillating chronic fluoride doses. Braz Dent J. 2010;21(1):32-7. doi: 10.1590/s0103-64402010000100005 10.1590/s0103-64402010000100005 20464318

[31] Shinoda H. Effect of long-term administration of fluoride on physico-chemical properties of the rat incisor enamel. Calcif Tissue Res. 1975;18(2):91-100. doi: 10.1007/BF02546229 10.1007/BF02546229 1148900

[32] Suckling G, Thurley DC, Nelson DG. The macroscopic and scanning electron-microscopic appearance and microhardness of the enamel, and the related histological changes in the enamel organ of erupting sheep incisors resulting from a prolonged low daily dose of fluoride. Arch Oral Biol. 1988;33(5):361-73. doi: 10.1016/0003-9969(88)90070-2 10.1016/0003-9969(88)90070-2 3190523

[33] Schours I, Massler M. The teeth. In: Farris E, Griffith J, editors. The rat in laboratory investigation. New York: Hafner Pub; 1963. p.104-60.

[34] Mihalaş E, Matricala L, Chelmuş A, Gheţu N, Petcu A, Paşca S. The role of chronic exposure to amoxicillin/clavulanic acid on the developmental enamel defects in mice. Toxicol Pathol. 2016;44(1):61-70. doi: 10.1177/0192623315610822 10.1177/0192623315610822 26534941

[35] Suzuki M, Bartlett JD. Rodent dental fluorosis model: extraction of enamel organ from rat incisors. Methods Mol Biol. 2019;1922:335-40. doi: 10.1007/978-1-4939-9012-2_30 10.1007/978-1-4939-9012-2_30 30838588

[36] Richards A. Nature and mechanisms of dental fluorosis in animals. J Dent Res. 1990;69 Spec No:701-5; discussion 721. doi: 10.1177/00220345900690S136 10.1177/00220345900690S136 2179332

[37] American Academy of Pediatrics Subcommittee on Management of Acute Otitis Media. Diagnosis and management of acute otitis media. Pediatrics. 2004;113(5):1451-65. doi: 10.1542/peds.113.5.1451 10.1542/peds.113.5.1451 15121972

[38] Kumazawa K, Sawada T, Yanagisawa T, Shintani S. Effect of single-dose amoxicillin on rat incisor odontogenesis: a morphological study. Clin Oral Investig. 2012;16(3):835-42. doi: 10.1007/s00784-011-0581-4 10.1007/s00784-011-0581-4 21717095

[39] Chachra D, Vieira AP, Grynpas MD. Fluoride and mineralized tissues. Crit Rev Biomed Eng. 2008;36(2-3):183-223. doi: 10.1615/critrevbiomedeng.v36.i2-3.40 10.1615/critrevbiomedeng.v36.i2-3.40 19740071

[40] Whitford GM. Intake and metabolism of fluoride. Adv Dent Res. 1994;8(1):5-14. doi: 10.1177/08959374940080011001 10.1177/08959374940080011001 7993560

[41] Bronckers AL, Lyaruu DM, DenBesten PK. The impact of fluoride on ameloblasts and the mechanisms of enamel fluorosis. J Dent Res. 2009;88(10):877-93. doi: 10.1177/0022034509343280 10.1177/0022034509343280 PMC3318083 19783795

[42] Ramesh M, Narasimhan M, Krishnan R, Aruna R, Kuruvilla S. The effect of fluorosis on human teeth under light microscopy: a cross-sectional study. J Oral Maxillofac Pathol. 2017;21(3):345-50. doi: 10.4103/jomfp.JOMFP_247_16 10.4103/jomfp.JOMFP_247_16 PMC5763854 29391706

[43] Chesa-Jimenez J, Peris JE, Torres-Molina F, Granero L. Low bioavailability of amoxicillin in rats as a consequence of presystemic degradation in the intestine. Antimicrob Agents Chemother. 1994;38(4):842-7. doi: 10.1128/AAC.38.4.842. 10.1128/aac.38.4.842 PMC284552 8031056

[44] Wuollet E, Laisi S, Salmela E, Ess A, Alaluusua S. Molar-incisor hypomineralization and the association with childhood illnesses and antibiotics in a group of Finnish children. Acta Odontol Scand. 2016;74(5):416-22. doi: 10.3109/00016357.2016.1172342 10.3109/00016357.2016.1172342 27140829

